# Ammonium bis­(salicyl­aldehyde thio­semi­car­ba­zonato)ferrate(III), a supra­molecular material containing low-spin Fe^III^

**DOI:** 10.1107/S2053229620006452

**Published:** 2020-05-29

**Authors:** Robyn E. Powell, Berthold Stöger, Christian Knoll, Danny Müller, Peter Weinberger, Petra J. van Koningsbruggen

**Affiliations:** a School of Engineering and Applied Science, Chemical Engineering and Applied Chemistry, Aston University, Aston Triangle, Birmingham B4 7ET, West Midlands, UK; b Aston Institute of Materials Research, Aston University, Birmingham B4 7ET, West Midlands, UK; c X-ray Center, Vienna University of Technology, Getreidemarkt 9, 1060 Vienna, Austria; d Institute of Applied Synthetic Chemistry, Vienna University of Technology, Getreidemarkt 9/163-01-3, 1060 Vienna, Austria

**Keywords:** ammonium, iron(III), crystal structure, low spin, order-disorder, maximum degree of disorder, MDO

## Abstract

The synthesis and crystal structure (100 K) of the title com­pound, ammonium bis­[salicyl­aldehyde thio­semi­car­ba­zon­ato(2−)-κ^3^ *O*,*N* ^1^,*S*]iron(III), NH_4_[Fe(C_8_H_7_N_3_OS)_2_], is reported. The asymmetric unit consists of an octa­hedral [Fe^III^(thsa)_2_]^−^ fragment, where thsa^2−^ is salicyl­aldehyde thio­semi­car­ba­zon­ate(2−), and an NH_4_^+^ cation. Each thsa^2−^ ligand binds *via* the thiol­ate S, the imine N and the phenolate O donor atoms, resulting in an Fe^III^S_2_N_2_O_2_ chromophore. The ligands are orientated in two perpendicular planes, with the O and S atoms in *cis* and the N atoms in *trans* positions. The Fe^III^ ion is in the low-spin state at 100 K. The crystal structure belongs to a category I order–disorder (OD) family. It is a polytype of a maximum degree of order (MDO). Fragments of the second MDO polytype lead to systematic twinning by pseudomerohedry.

## Abstract 2

The Fe^III^S_2_N_2_O_2_ chromophore of ammonium bis­[salicyl­aldehyde thio­semi­car­ba­zon­ato(2−)]iron(III) contains two *O*,*N*,*S*-donating salicyl­aldehyde thio­semi­car­ba­zon­ate(2−) ligands in perpendicular planes, with the O and S atoms in *cis* and the N atoms in *trans* positions. The Fe^III^ ion is in the low-spin state at 100 K. Systematic twinning by metric pseudomerohedry is explained by application of the order–disorder (OD) theory.

## Introduction   

The study of the coordination chemistry of thio­semi­car­ba­zones is an attractive research area. Thio­semicarbazones display a wide range of pharmacological uses based, for example, on their anti­neoplastic, anti­bacterial, anti­viral and anti­fungal activities (Beraldo & Gambino, 2004 ▸; Yemeli Tido *et al.*, 2010 ▸). This pharmacological action is often related to coordination of the thio­semicarbazone to metal ions (Farrell, 2002 ▸). On the other hand, the magnetic properties of iron(III) com­pounds of thio­carbazone derivatives have attracted attention, particularly as switching behaviour was displayed for iron(III) bound to particular salicyl­aldehyde thio­semicarbazone derivatives (van Koningsbruggen *et al.*, 2004 ▸; Phonsri *et al.*, 2017 ▸; Powell *et al.*, 2014 ▸, 2015 ▸; Yemeli Tido, 2010 ▸; Zelentsov *et al.*, 1973 ▸, Ryabova *et al.*, 1978 ▸, 1981*a* ▸,*b* ▸, 1982 ▸; Floquet *et al.*, 2003 ▸, 2006 ▸, 2009 ▸; Li *et al.*, 2013 ▸).

This type of magnetic inter­conversion between the low-spin (*S* = 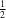) and high-spin (*S* = 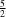) state in Fe^III^ (3*d* ^5^) systems has now been found to be triggered by a change in temperature or pressure, or by light irradiation (Hayami *et al.*, 2000 ▸, 2009 ▸; van Koningsbruggen *et al.*, 2004 ▸) and may be used in a functional way in research and technology (Létard *et al.*, 2004 ▸; Gütlich *et al.*, 2004 ▸; Gütlich & Goodwin 2004 ▸; van Koningsbruggen *et al.*, 2004 ▸; Nihei *et al.*, 2007 ▸; Halcrow, 2013 ▸; Harding *et al.*, 2016 ▸).

In recent years, particular inter­est has focused on Fe^III^ com­plexes of substituted derivatives of *R*-salicyl­aldehyde 4*R*′-thio­semicarbazone (Powell *et al.*, 2014 ▸, 2015 ▸; Yemeli Tido, 2010 ▸; Floquet *et al.*, 2003 ▸, 2006 ▸, 2009 ▸; Li *et al.*, 2013 ▸) for generating Fe^III^ spin crossover. In solution, free *R*-salicyl­aldehyde 4*R*′-thio­semicarbazone (H_2_ *L*) exists in two tautomeric forms, *i.e*. the thione and thiol forms, as illustrated in Scheme 1. The chemistry of the Fe^III^ com­pounds is rather com­plicated as it is possible for the tridentate *R*-salicyl­alde­hyde 4*R*′-thio­semicarbazone ligand (H_2_ *L*) to exist in tautomeric forms; moreover, the ligand may also be present in its neutral, anionic or dianionic form. However, the formation of a particular type of Fe^III^ com­plex unit, *i.e.* neutral, monocationic or monoanionic, can be achieved by tuning the degree of deprotonation of the ligand through pH variation of the reaction solution during the synthesis (Powell *et al.*, 2014 ▸, 2015 ▸; Powell, 2016 ▸; Yemeli Tido, 2010 ▸; Floquet *et al.*, 2009 ▸). We have been particularly skilled in preparing anionic Fe^III^ com­plexes of the general formula (cation^+^)[Fe(*L* ^2−^)_2_]·*x*(solvent), for which it became evident that the electronic state of the Fe^III^ ion is dependent on the nature of the counter-ion, the nature and degree of solvation and the nature of the *R*,*R*′-substituted ligands (Powell *et al.*, 2014 ▸, 2015 ▸; Powell, 2016 ▸; Yemeli Tido, 2010 ▸).
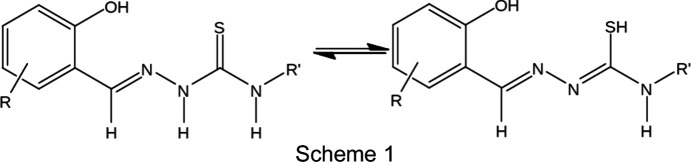


We report here a novel Fe^III^ com­pound, ammonium bis­[salicyl­aldehyde thio­semi­car­ba­zon­ato(2−)-κ^3^ *O*,*N* ^1^,*S*]iron(III), (I) (see Scheme 2), containing two dianionic tridentate ligands, *i.e*. salicyl­aldehyde thio­semi­car­ba­zon­ate(2−), abbreviated as thsa^2−^, whose structure was determined at 100 K. Ryabova *et al.* (1981*a* ▸) reported the crystallographic data of the related com­pound Cs[Fe(thsa)_2_] at 103 and 298 K, which contains Fe^III^ in the high-spin electronic state (*S* = 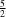). The main difference between NH_4_[Fe(thsa)_2_] and Cs[Fe(thsa)_2_] is the associated outer-sphere cation. This article describes that the variation in the cation leads to a modification of the structure of the Fe^III^ com­pound, also changing the crystal packing, and being responsible for the Fe^III^ in the present NH_4_[Fe(thsa)_2_] com­pound exhibiting the low-spin electronic state (*S* = 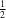).

Compound (I) systematically crystallizes as twins. The twinning will be inter­preted in the light of order–disorder (OD) theory (Dornberger-Schiff & Grell-Niemann, 1961 ▸). The OD theory was created in the 1950s to explain the common occurrence of polytypism and stacking faults. It has since been developed into a com­prehensive theory of local/partial symmetry. According to OD theory, if a structure is com­posed of layers and the layers are related by partial symmetry that is not valid for the whole structure, then the stacking becomes ambiguous. This means that the layers can be arranged in different ways, which are nevertheless all locally equivalent. Owing to the short range of inter­atomic inter­actions, these different stacking arrangements are also energetically equivalent. Consequently, OD structures often feature stacking faults. In OD twins, such as the title com­pound, the stacking faults lead to domains with different spacial orientations and are sporadic, *i.e.* the resulting domains are macroscopic and do not diffract coherently.
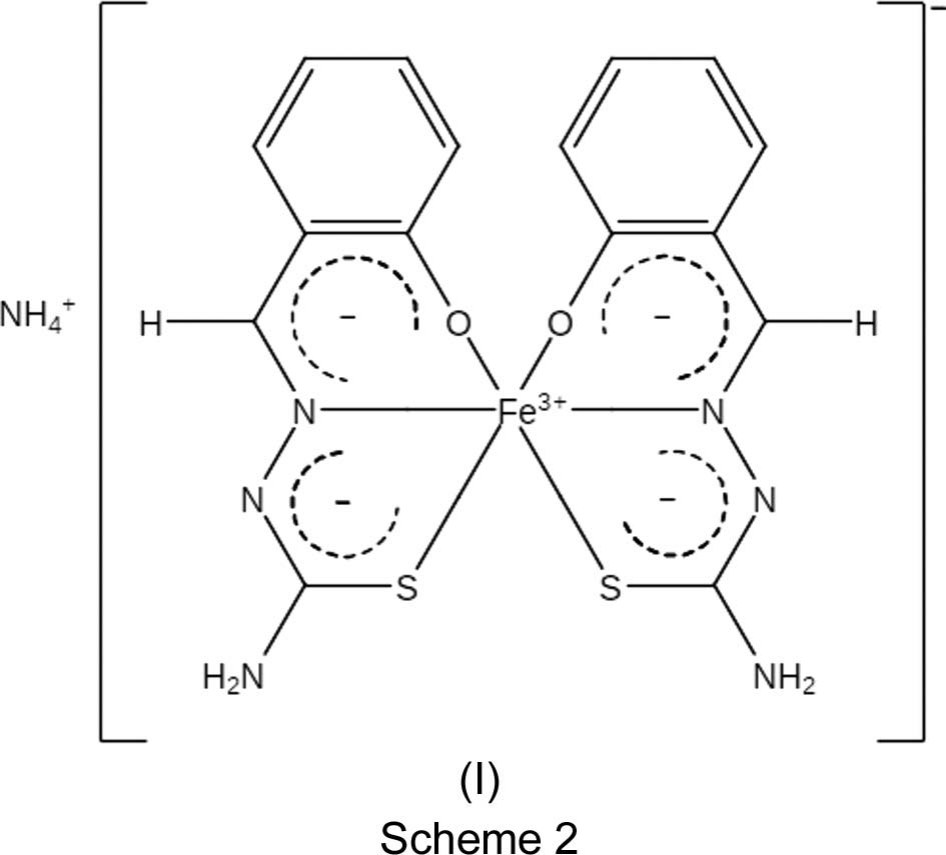


## Experimental   

### Spectroscopic measurements   

Room-temperature IR spectra within the range 4000–400 cm^−1^ were recorded on a PerkinElmer FT–IR spectrometer Spectrum RXI using KBr pellets. Variable-temperature FT–IR spectra were measured with the attenuated total reflectance (ATR) technique using a PerkinElmer spectrum 400 with a Harrick diamond ATR equipped with a thermostatable temperature-control device. ^1^H and ^13^C{^1^H} NMR spectra were recorded on a Bruker 200 spectrometer with a broadband probe head. All NMR chemical shifts are reported in ppm; ^1^H and ^13^C shifts are established on the basis of the residual solvent resonance.

### Synthesis and crystallization   

The synthesis of H_2_thsa was carried out according to the general procedure described by Yemeli Tido (2010 ▸). Salicyl­aldehyde (49 mmol, 5.98 g) was dissolved in ethanol (80 ml) with constant stirring, and was added to a solution of thio­semicarbazide (49 mmol, 3.68 g) in ethanol (40 ml). The corresponding mixture was refluxed for 120 min. The resulting solution was cooled to room temperature and the solid isolated by filtration, washed with ether and dried in a vacuum for 2 d (yield: 7.75 g, 39.7 mmol, 81.0%; m.p. 491 K). H_2_thsa is soluble in methanol, acetone and dimethyl sulfoxide (DMSO). ^1^H NMR (200 MHz, DMSO-*d*_6_): δ (ppm) 11.39 (*s*, 1H, NH), 9.84 (*s*, 1H, OH), 8.40 (*s*, 1H, CH), 8.10 (*s*, 1H, *o*-ArCH), 7.87 (*d*, *J* = 8.8 Hz, 2H, NH_2_), 7.19 (*t*, *J* = 7.7 Hz, 1H, *p*-ArCH), 6.92–6.71 (*m*, 2H, *m*-ArCH). ^13^C NMR (50 MHz, DMSO-*d*_6_): δ (ppm) 177.76 (C=S), 156.52 (C—OH), 140.16 (C=N), 131.31 (ArCH), 126.99 (ArCH), 120.33 (ArCH), 119.48 (ArCH), 116.22 (ArCH). IR (cm^−1^, KBr): 3445 (νOH), 3175 (νNH), 3319 (νNH_2_), 1616 (νC=N), 1540–1603 (νC=C), 1266 (νC—N), 1111 (νC=S).

For the synthesis of NH_4_[Fe(thsa)_2_], (I), Fe(*p*-CH_3_C_6_H_4_SO_3_)_3_·6H_2_O (1.0 mmol, 0.68 g) was dissolved in methanol (5 ml). H_2_thsa (1.0 mmol, 0.20 g) was dissolved in methanol (25 ml) with the addition of NH_4_OH (20 ml, 35 wt% in water). To this mixture, the methano­lic solution of the Fe^III^ salt was added dropwise with constant stirring. The resulting dark-green solution was stirred and heated to 353 K for approximately 10 min. The solution was then allowed to stand at room temperature until crystals had formed. The dark-green microcrystals were isolated by filtration and dried (yield: 0.10 g, 0.22 mmol, 21.7%). Elemental analysis found/calculated (%) for C_16_H_18_FeN_7_O_2_S_2_: C, 41.47/41.47, H 3.90/3.94, N 20.99/21.30, O 8.05/6.95, S 13.87/13.93. IR (cm^−1^, KBr): 3472, 3240 (νNH), 3016 (νNH_2_), 1595 (νC=N), 1546–1509 (νC=C ring), 1278 (νC—O), 1203 (νN—N), 1027 (νC—N), 756 (νC—S).

### Refinement   

Crystal data, data collection and structure refinement details are summarized in Table 1 ▸. The crystal was modelled as twinned by reflection at (100). The positions of the H atoms on the amine N atoms were located in difference Fourier maps and were refined with restrained N—H distances of 0.87 (2) Å. The NH_4_^+^ cation is disordered around a pseudo-twofold axis. The ammonium N atom was refined as disordered about two positions (N7 and N7′). The sum of the occupancy parameters was constrained to 1. The atomic displacement parameters (ADPs) were constrained to the same value, resulting in a significant decrease of the estimated standard uncertainty on the occupancy parameters. Even though residual electron density in the difference Fourier maps could be attributed to the H atoms of the disordered ammonium positions, a reliable refinement was not possible. The ammonium H atoms were therefore ultimately omitted from the refinement. Other H atoms were included in the refinement in calculated positions and allowed to ride on their parent atoms.

## Results and discussion   

### Crystal structure   

**Figure 1:**
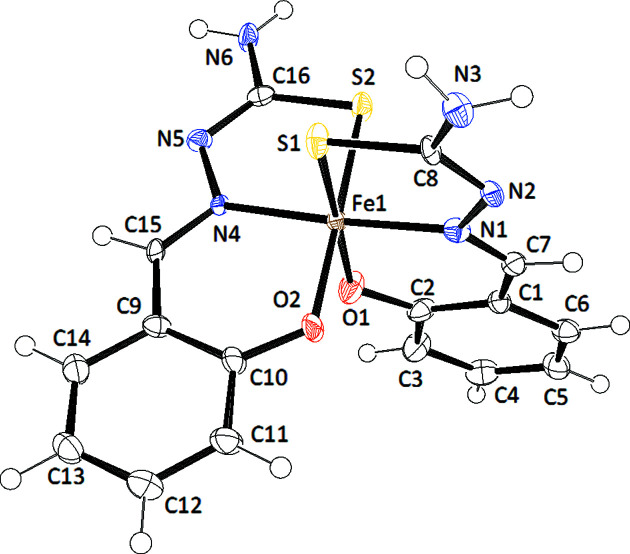
The mol­ecular structure and atom-numbering scheme for NH_4_[Fe(thsa)_2_], (I). The N atom of the NH_4_^+^ cation has been omitted for clarity. Displacement ellipsoids are drawn at the 50% probability level.

The structure of NH_4_[Fe(thsa)_2_], (I) (Fig. 1 ▸), was determined at 100 K and was found to crystallize in the monoclinic space group *P*2_1_/*n*. The asymmetric unit consists of one for­mula unit, *i.e.* NH_4_[Fe(thsa)_2_], with no atom on a special position. The Fe^III^ cation is coordinated by two dianionic *O*,*N*,*S*-tridentate chelating thsa^2−^ ligands, displaying a dis­torted octa­hedral Fe^III^O_2_N_2_S_2_ geometry. Selected geometric parameters are listed in Table 2 ▸. The twofold deprotonated ligands are coordinated to the Fe^III^ atom *via* the phenolate O, thiol­ate S and imine N atoms. These donor–Fe^III^ bonds are located in two perpendicular planes, with the O and S atoms in *cis* positions, whereby the S1—Fe—S2 and O1—Fe—O2 angles are 92.07 (3) and 88.53 (12)°, respectively. In addition, the N atoms are situated in *trans* positions, which is evidenced by the N1—Fe—N4 bond angle of 176.71 (11)°. The FeO_2_N_2_S_2_ coordination core is distorted; the Fe–donor atom distances fall within the range expected for Fe^III^ in the low-spin state (van Koningsbruggen *et al.*, 2004 ▸).

The incorporation of a monovalent NH_4_^+^ cation could be corroborated by variable-temperature FT–IR spectroscopy, which revealed the sharpening of the N—H stretching vibrational mode of the NH_4_^+^ cation at 3240 cm^−1^ upon cooling from ambient temperature to 173 K, which is in line with the freezing of the rotation of the NH_4_^+^ cation in the cavity.

The presence of the trivalent iron cation is supported by the coordination of two doubly deprotonated ligands to the Fe^III^ ion. In addition, the presence of both dianionic ligands is confirmed by the C—S, C—N and N—N bond lengths (Table 2 ▸) obtained for NH_4_[Fe(thsa)_2_], which show characteristics of a bond order between single and double bonds. Ryabova *et al.* (1981*a* ▸) reported the structure of the related high-spin com­pound Cs[Fe(thsa)_2_] at 103 and 298 K, which crystallizes in the space group *Pna*2_1_ with an asymmetric unit consisting of a Cs^+^ cation and an [Fe(thsa)_2_]^−^ anionic unit. The C—S, C—N and N—N bond lengths [at 103 K: C—S = 1.749 (9) and 1.761 (9) Å; C—N = 1.314 (10) and 1.303 (11) Å; N—N = 1.371 (11) and 1.380 (11) Å; at 298 K: C—S = 1.743 (14) and 1.775 (17) Å; C—N = 1.281 (19) and 1.281 (19) Å; N—N = 1.393 (18) and 1.412 (18) Å] reported by Ryabova *et al.* (1981*a* ▸) for Cs[Fe(thsa)_2_], correspond to the bond lengths for NH_4_[Fe(thsa)_2_], (I), at 100 K.

**Figure 2:**
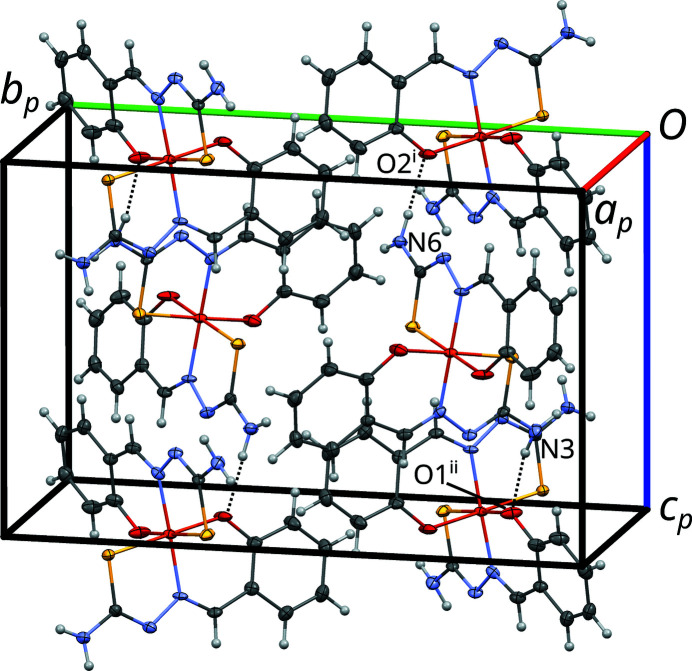
A projection showing the unit cell of NH_4_[Fe(thsa)_2_], (I). The N atom of the NH_4_^+^ cation has been omitted for clarity. Displacement ellipsoids are drawn at the 50% probability level. Dashed lines indicate hydrogen bonds. [Symmetry codes: (i) *x* − 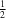, −*y* + 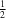, *z* − 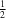; (ii) *x* − 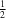, −*y* + 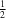, *z* + 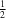.]

The hydrogen-bonding inter­actions of NH_4_[Fe(thsa)_2_] are listed in Table 3 ▸ and are displayed in Fig. 2 ▸. The terminal N atoms of the tridentate ligands (N3 and N6), form N6—HN62⋯O2^i^ and N3—HN32⋯O1^ii^ contacts with the phe­nol­ate donor atoms O1 and O2, respectively. In this manner, successive Fe^III^ entities are linked in the *c* direction. The NH_4_^+^ cations are distributed in between the layers of the Fe^III^ entities, with alternate separations of the N atoms of the NH_4_^+^ cation of 3.915 (7) [at (*x* + 1, *y*, *z*), denoted iii] and 4.547 (7) Å [at (*x* + 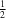, −*y* + 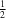, *z* + 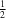), denoted iv] in the *a* direction. The Fe^III^⋯Fe^III^ separations in the present com­pound are 8.4393 (8) Å for Fe^III^⋯Fe^IIIiii^ and 9.7134 (13) Å for Fe^III^⋯Fe^IIIv^ [symmetry code: (v) −*x*, −*y* + 1, −*z* + 1]. The Fe^III^ units in NH_4_[Fe(thsa)_2_] are linked by hydrogen-bonding inter­actions between the corresponding phenolate O and amino N atoms of the Fe^III^ units.

The embedding of the NH_4_^+^ cation is, therefore, essentially different from that of the Cs^+^ cation in Cs[Fe(thsa)_2_] at 103 and 298 K, where the nearest-neighbour coordination sphere of the Cs^+^ cation is constituted by O, N and C atoms, which form a seven-pointed polyhedron with Cs–(ligand donor atom) separations between 3.06 and 3.82 Å (Ryabova *et al.*, 1981*a* ▸). This feature shows some similarity with the Cs^+^ cation in Cs[Fe(5-Br-thsa)_2_] (Powell *et al.*, 2015 ▸) that is at the centre of an irregular seven-donor-atom polyhedron, the donor atoms of which originate from symmetry-related equivalents of both symmetry-independent 5-bromo­salicyl­aldehyde thio­semi­car­ba­zon­ate(2−) (5-Br-thsa) ligands. Several donor atoms coordinated to the Fe^III^ atom of Cs[Fe(5-Br-thsa)_2_] form inter­actions with the Cs^+^ cation in the second coordination sphere; this is likely to modulate the electron density of the Fe–(donor atom) bonds and hence influence the electronic state of the Fe^III^ cation. The latter is also prone to be affected by the assembly of Fe^III^ units in the unit cell. The presence of the Br substituent on the salicyl­aldehyde group of the ligand in Cs[Fe(5-Br-thsa)_2_] is a factor in determining the crystal packing, as the Br substituent of one Cs[Fe(5-Br-thsa)_2_] unit provides a hydrogen-bonding inter­action with an amino group of a neighbouring Fe^III^ unit, creating ring systems.

Clearly, the variation in cation, ligand substituents and crystal packing is related to the spin state of Fe^III^ being high-spin in Cs[Fe(thsa)_2_] at 103 and 298 K (Ryabova *et al.*, 1981*a* ▸), low-spin in Cs[Fe(5-Br-thsa)_2_] at 293 K (Powell *et al.*, 2015 ▸) and low-spin in the present NH_4_[Fe(thsa)_2_] at 100 K. Variable-temperature magnetic susceptibility measurements (10–300 K) confirm that the Fe^III^ ion in NH_4_[Fe(thsa)_2_] remains in the low-spin state over this temperature range (Powell, 2016 ▸). In addition, within this family of cation(+) bis­[*R*-salicyl­aldehyde 4*R*′-thio­semi­car­ba­zon­ato(2−)]ferrate(III) salts, the presence of particular solvent mol­ecules may further affect the crystal packing, with the associated inter­molecular effects influencing the electronic structure of Fe^III^, *e.g.* leading to a low-spin state of Fe^III^ in Cs[Fe(*L*)_2_]·CH_3_OH [*L* = 3-eth­oxy­salicyl­aldehyde 4-methyl­thio­semi­car­ba­zon­ate(2−)] at 100 K (Powell *et al.*, 2014 ▸). Our further studies of members of this Fe^III^ family may shed more light on how the spin state of Fe^III^ may be tuned in these systems.

### Systematic twinning and OD theory   

Even though NH_4_[Fe(thsa)_2_] crystallizes in the monoclinic space group *P*2_1_/*n*, the lattice is metrically virtually ortho­rhom­bic primitive [*oP*, β = 90.052 (4)°]. The crystals are systematically twinned by the additional symmetry of the lattice with respect to the 2/*m* point symmetry of the crystal. Thus, the twin law com­prises the operations {2_[100]_, *m*_[100]_, 2_[001]_, *m*_[001]_} and the twin point group (Nespolo, 2004 ▸) is 2′/*m*′2/*m*2′/*m*′. Since the reflections of both domains overlap nearly perfectly (twin index 1, twin obliquity ∼0), the twinning can be classified as being by *metric pseudomerohedry* (Nespolo & Ferraris, 2000 ▸).

A higher point symmetry of the lattice com­pared to the point symmetry of the crystal is often associated with twinning. However, it is not a sufficient precondition for its existence. In fact, many polar structures do not form twins by inversion despite inversion being an intrinsic symmetry of any lattice. One common and often overlooked cause of twinning is partial symmetry, which may lead to a twin inter­face that is locally equivalent to the twin individuals. The order–disorder (OD) theory (Dornberger-Schiff & Grell-Niemann, 1961 ▸) was introduced in the 1950s to deal precisely with these kinds of structures.

**Figure 3:**
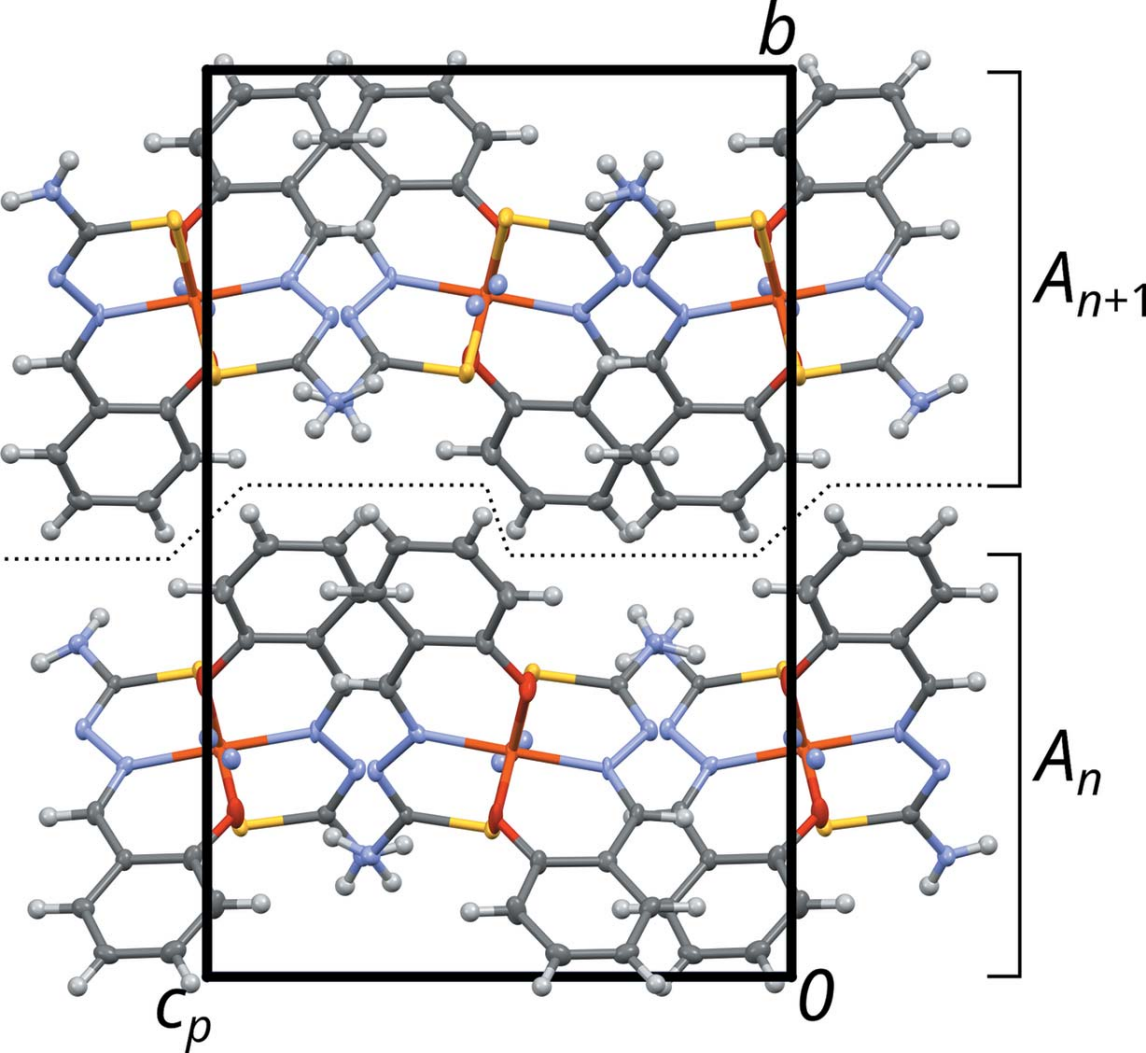
The crystal structure of NH_4_[Fe(thsa)_2_], viewed down [100]. The names of the OD layers are indicated to the right and a dotted line indicates the inter­face between the OD layers, which in this case is not planar.

**Figure 4:**
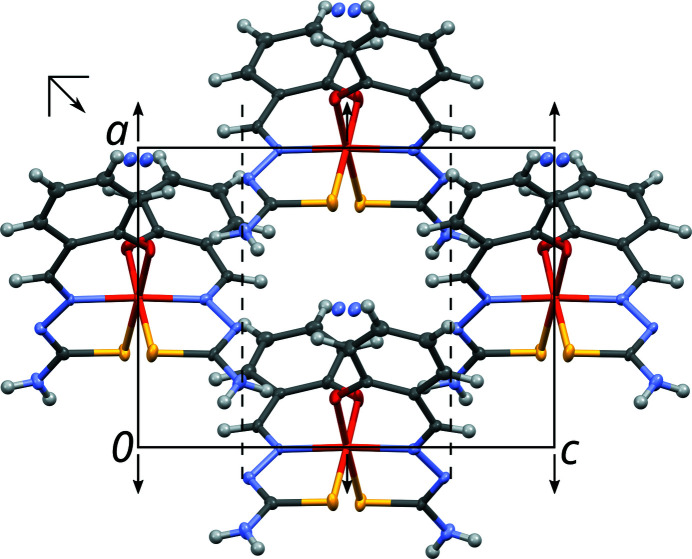
A layer in the crystal structure of NH_4_[Fe(thsa)_2_] projected on the layer plane (010). Symmetry elements are represented by the usual graphical symbols. The indicated unit cell corresponds to the standard origin choice of the *P*2(*n*)*a* layer group (on 2_[100]_), not of the overall crystal structure.

In the light of OD theory, the crystal structure of NH_4_[Fe(thsa)_2_] can be decom­posed into OD layers *A_n_* (*n* being a sequential number) parallel to (010), which, in this case, also correspond to layers in the crystallochemical sense (Fig. 3 ▸). The crucial point of an OD structure is that partial symmetry operations relate individual layers, yet need not be valid for the whole structure. In the case of NH_4_[Fe(thsa)_2_], the *A_n_* layers possess (idealized) *P*2(*n*)*a* symmetry (Fig. 4 ▸). In this layer-group notation, which is commonly used in the OD literature, the parentheses indicate the direction missing translational symmetry. The [Fe(thsa)_2_]^−^ ions are located on the twofold axes of the *A_n_* layers, whereas the ammonium ions are disordered about these axes.

The set of partial symmetry operations of any possible stacking of NH_4_[Fe(thsa)_2_] is described by the OD groupoid family symbol (Dornberger-Schiff & Grell-Niemann, 1961 ▸).

*P* 2 (*n*) *a*

*n*_2,*r*_ 2_2_ 2_*r*–1_

OD groupoid families are the analogue of space group types in classical crystallography. They abstract from metric parameters and additionally of the particular stacking. The first line of the symbol indicates the layer symmetry, the second line one set of operations relating adjacent layers. Since the intrinsic translations are not limited to those found in space groups, a generalization of the Hermann-Mauguin notation is used. For example, *n*_2,*r*_ represents a glide reflection with the intrinsic translation **b**/2 + *r* **c**/2, whereby *r* is one of the metric parameters the OD groupoid family abstracts from.

Owing to the partial symmetry, layers can be arranged in different ways while keeping pairs of adjacent layers geometrically equivalent. These stacking possibilities can be enumerated using the *NFZ* relationship (Ďurovič, 1997 ▸), which reads as *Z* = *N*/*F* = [*G_n_*:*G_n_*∩*G_n_*_+1_]. *G_n_* = *P*1(1)*a* is the group of operations of the *A_n_* layer that do not invert *A_n_* with respect to the stacking direction. Since the *a*-glide planes of adjacent layers do not overlap, *G_n_*∩*G_n_*_+1_ = *P*1(1)1. The possible layer arrangements are determined by coset decom­position of the latter in the former. In other words, given the *A_n_* layer, the adjacent *A_n_*_+1_ layer can be placed in *Z* = [*P*1(1)*a*:*P*1(1)1] = 2 ways, which are related by the *a*-glide reflection of the *A_n_* layer.

Of the infinity of the thus obtained locally equivalent polytypes, a finite number is especially simple in the sense that they cannot be decom­posed into fragments of even simpler polytypes. In these polytypes, which are said to be of a maximum degree of order (MDO), not only pairs but also triples, quadruples and generally *n*-tuples of adjacent layers are equivalent (for a more rigorous definition, see Dornberger-Schiff, 1982 ▸). Polytypes of the MDO type play a special role in OD theory, because all other polytypes can be decom­posed into fragments of MDO polytypes. Moreover, experience shows that ordered bulk polytypes are in most cases of the MDO type. The OD family of NH_4_[Fe(thsa)_2_] contains two MDO polytypes:

MDO_1_: *P*2/*b*11, **b** = 2**b**_0_ + *r* **c**

**Figure 5:**
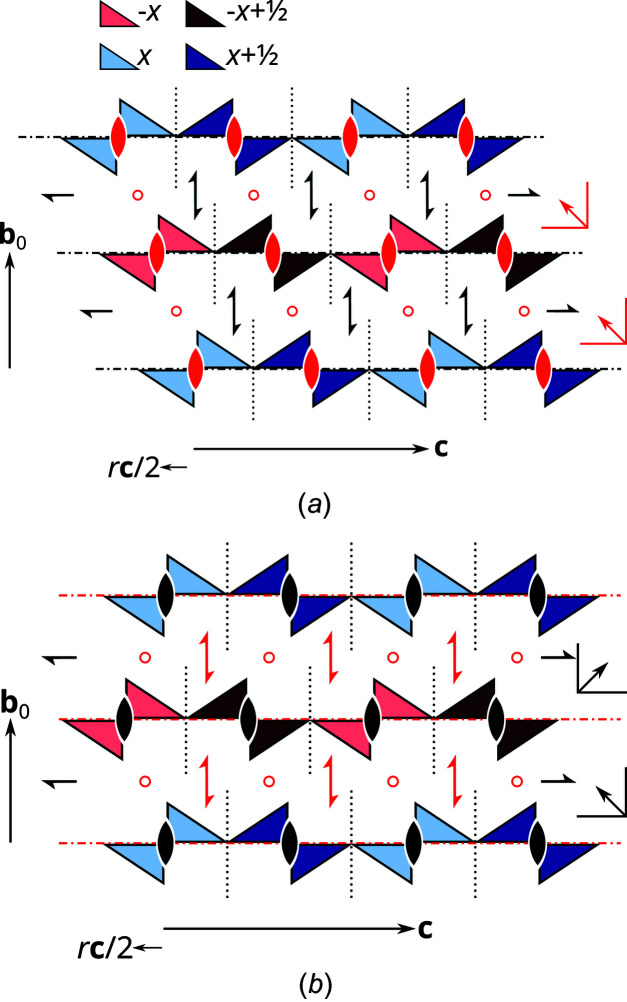
Schematic representation of the (top) MDO_1_ and (bottom) MDO_2_ polytypes of NH_4_[Fe(thsa)_2_]. [Fe(thsa)_2_]^−^ ions are represented by pairs of triangles which are blue on one side and red on the other side. Darker colours indicate a translation by **a**/2. The geometrical elements of partial sym­metry operations of layers and those relating adjacent layers are represented by the usual graphical symbols for symmetry elements. Screw axes and glide planes with nonstandard intrinsic translations are represented using the symbols for the 2_1_ and *n* symmetry elements. Symbols of operations that are valid for the whole polytype are shown in red.

MDO_2_: *P*2_1_/*n*, **b** = 2**b**_0_

where **b**_0_ is the vector perpendicular to the layer lattices with the length of one layer width. Both MDO polytypes are shown schematically in Fig. 5 ▸. The twin individuals of NH_4_[Fe(thsa)_2_] correspond to the MDO_2_ polytype. A fragment of the MDO_1_ polytype is located at the twin inter­face.

Thus, the OD theory plausibly explains the formation of the observed twins, as the twin inter­face is geometrically and, if inter­actions over one layer width are ignored, also energetically equivalent to the twin individuals. Moreover, it explains the pseudo-*oP* metrics of the lattice. Such a metric pseudo-symmetry has often been considered as ‘accidental’. However, here it is clearly very much intrinsic to the structure family.

Finally, it should be noted that an OD description is usually based on a certain degree of idealization. Ordered polytypes are desymmetrized with respect to the ideal description (Ďurovič, 1979 ▸). Indeed, in the actual MDO_2_ polytypes of NH_4_[Fe(thsa)_2_], the symmetry of the *A_n_* layers is reduced by an index of 2 from *P*2(*n*)*a* to *P*1(*n*)1. Accordingly, the site symmetry of the [Fe(thsa)_2_]^−^ ion is reduced from 2 to 1. Moreover, the unit-cell parameters deviate slightly from ortho­rhom­bic metrics [β = 90.052 (4)°, according to single-crystal diffraction]. Finally, the desymmetrization is also observed by a splitting of the single disordered ammonium position into two independent positions, which are now not forcibly disordered in a 1:1 manner. Indeed, the ratio of the occupancies of both positions refines to 52.7 (9):47.3 (9). However, collectively the deviations from the idealized partial symmetry are minute and the OD description can be considered as correct.

## Supplementary Material

Crystal structure: contains datablock(s) I, global. DOI: 10.1107/S2053229620006452/ky3195sup1.cif

Structure factors: contains datablock(s) I. DOI: 10.1107/S2053229620006452/ky3195Isup2.hkl

CCDC reference: 2003812

## Figures and Tables

**Table 1:** Experimental details

Crystal data	
Chemical formula	(NH_4_)[Fe(C_8_H_7_N_3_OS)_2_]
*M*_r_	460.34
Crystal system, space group	Monoclinic, *P*2_1_/*n*
Temperature (K)	100
*a*, *b*, *c* (Å)	8.4393 (8), 18.2444 (17), 11.7635 (11)
β (°)	90.052 (4)
*V* (Å^3^)	1811.2 (3)
*Z*	4
Radiation type	Mo *K*α
μ (mm^−1^)	1.09
Crystal size (mm)	0.60 × 0.36 × 0.18

Data collection	
Diffractometer	Bruker Kappa APEXII CCD
Absorption correction	Multi-scan (*SADABS*; Bruker, 2012 ▸)
*T*_min_, *T*_max_	0.601, 0.746
No. of measured, independent and observed [*I* > 2σ(*I*)] reflections	62618, 5355, 4839
*R*_int_	0.041
(sin θ/λ)_max_ (Å^−1^)	0.708

Refinement	
*R*[*F* ^2^ > 2σ(*F* ^2^)], *wR*(*F* ^2^), *S*	0.042, 0.110, 1.10
No. of reflections	5355
No. of parameters	274
No. of restraints	4
H-atom treatment	H atoms treated by a mixture of independent and constrained refinement
Δρ_max_, Δρ_min_ (e Å^−3^)	0.93, −0.74

Computer programs: *APEX2* (Bruker, 2012 ▸), *SAINT-Plus* (Bruker, 2012 ▸), *SUPERFLIP* (Palatinus & Chapuis, 2007 ▸), *SHELXL2018* (Sheldrick, 2015 ▸) and *ORTEP-3* (Farrugia, 2012 ▸).

**Table 2:** Selected geometric parameters (Å, °)

Fe1—N1	1.937 (3)	S1—C8	1.745 (3)
Fe1—O1	1.941 (3)	S2—C16	1.747 (3)
Fe1—N4	1.944 (3)	N1—C7	1.295 (4)
Fe1—O2	1.952 (3)	N1—N2	1.398 (4)
Fe1—S1	2.2369 (10)	N4—C15	1.293 (4)
Fe1—S2	2.2377 (10)	N4—N5	1.406 (4)
			
N1—Fe1—O1	93.08 (11)	C16—S2—Fe1	95.57 (12)
N1—Fe1—N4	176.71 (11)	C2—O1—Fe1	126.5 (2)
O1—Fe1—N4	88.73 (11)	C10—O2—Fe1	126.4 (2)
N1—Fe1—O2	88.97 (11)	C7—N1—Fe1	125.7 (2)
O1—Fe1—O2	88.53 (12)	C8—N2—N1	113.4 (3)
N4—Fe1—O2	93.83 (11)	C15—N4—Fe1	125.0 (2)
N1—Fe1—S1	86.44 (9)	C16—N5—N4	113.7 (3)
O1—Fe1—S1	177.74 (10)	C2—C1—C7	122.4 (3)
N4—Fe1—S1	91.85 (9)	O1—C2—C1	124.5 (3)
O2—Fe1—S1	89.26 (10)	N1—C7—C1	126.9 (3)
N1—Fe1—S2	91.60 (9)	N2—C8—S1	124.7 (2)
O1—Fe1—S2	90.15 (9)	C10—C9—C15	124.2 (3)
N4—Fe1—S2	85.65 (9)	O2—C10—C9	123.6 (3)
O2—Fe1—S2	178.59 (10)	N4—C15—C9	126.4 (3)
S1—Fe1—S2	92.07 (3)	N5—C16—S2	124.0 (3)
C8—S1—Fe1	94.63 (11)		

**Table 3:** Hydrogen-bond geometry (Å, °)

*D*—H⋯*A*	*D*—H	H⋯*A*	*D*⋯*A*	*D*—H⋯*A*
N3—HN31⋯O1^i^	0.87 (2)	2.03 (3)	2.879 (4)	164 (6)
N6—HN62⋯O2^ii^	0.88 (2)	1.96 (2)	2.834 (4)	178 (5)

Symmetry codes: (i) 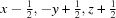; (ii) 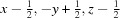.
